# 3D-Printed PLA Filaments Reinforced with Durian (*Durio zibethinus*) Husk-Derived Carboxymethyl Cellulose for Methylene Blue Removal

**DOI:** 10.3390/polym18141707

**Published:** 2026-07-11

**Authors:** Kawisara Sirichaicharoenkol, Anchan Khankhuean, Weekit Sirisaksoontorn, Chainarong Sakulthaew, Ut Dong Thach, Philip Anggo Krisbiantoro, Kevin C.-W. Wu, Chih-Feng Huang, Tongsai Jamnongkan

**Affiliations:** 1 Department of Chemistry, Faculty of Science, Kasetsart University, Bangkok 10900, Thailand; kawisara.siric@live.ku.th (K.S.); fsciwks@ku.ac.th (W.S.); 2 Department of Fundamental Science and Physical Education, Faculty of Science at Sriracha, Kasetsart University, Chonburi 20230, Thailand; anchan.kh@ku.th; 3 Department of Veterinary Nursing, Faculty of Veterinary Technology, Kasetsart University, Bangkok 10900, Thailand; cvtcns@ku.ac.th; 4 Research Group in Pharmaceutical and Biomedical Sciences, Faculty of Pharmacy, Ton Duc Thang University, Ho Chi Minh City 700000, Vietnam; thachutdong@tdtu.edu.vn; 5 Department of Applied Chemistry, Graduate School of Engineering, Tohoku University, Sendai 980-8579, Japan; anggokrisbiantoro@gmail.com (P.A.K.); kevinwu@ntu.edu.tw (K.C.-W.W.); 6 Department of Chemical Engineering, National Taiwan University, Taipei 10617, Taiwan; 7 Department of Chemical Engineering and Materials Science, Yuan Ze University, Taoyuan 320, Taiwan; 8 Department of Chemical Engineering, Chung Yuan Christian University, Taoyuan 320, Taiwan; 9 Department of Chemical Engineering, i-Center for Advanced Science and Technology (i-CAST), National Chung Hsing University, Taichung 40227, Taiwan; huangcf@dragon.nchu.edu.tw

**Keywords:** 3D printing, polylactic acid, durian husk, carboxymethyl cellulose, biocomposite, adsorption, methylene blue

## Abstract

Although polylactic acid (PLA) filaments are currently the leading biodegradable polymer in three-dimensional (3D) printing due to their excellent printability, good mechanical properties, and environmental friendliness, they possess low thermal resistance and limited flexibility, which restricts their use in applications requiring enhanced material performance. PLA filaments were reinforced with carboxymethyl cellulose (CMC) derived from durian husk using a single-screw extrusion technique to produce PLA/CMC filaments. CMC was obtained via carboxymethylation of durian husk-derived cellulose using monochloroacetic acid (MCA) in the presence of NaOH. The incorporation of CMC improved the thermal and mechanical properties of the PLA filaments. The presence of carboxylate (–COO–) groups from CMC makes the filaments promising as an adsorbent for a dye pollutant, namely, methylene blue (MB). Adsorption tests showed that the performance of PLA/CMC filaments for MB removal was influenced by the CMC loading, with 0.1 wt.% CMC (PLA/CMC-30 (0.1)) being the optimal one for achieving a higher adsorption rate. Over the filaments, the removal of MB followed a pseudo-first-order kinetic model, with a *k*_1_ of 0.020 min^−1^. Overall, this work demonstrates that PLA/CMC filaments derived from agricultural waste offer a sustainable and multifunctional material with enhanced performance for environmental remediation applications.

## 1. Introduction

Currently, 3D printing, also known as additive manufacturing [1], has attracted significant attention across various fields, including aerospace [2], automotive industry [3], food industry [4], medical and healthcare [5], architecture [6], and the textile industry [7] due to its ability to enable rapid prototyping, fabricate complex geometries, reduce production costs, and offer an easy and flexible manufacturing process. Furthermore, recent studies have also developed 3D printed composite materials as functional water treatment devices, offering materials for the adsorption and filtration of pollutants [8]. In the field of material synthesis, these advantages are further complemented by the capability of 3D printing to precisely control material composition and structure, thereby facilitating the development of advanced functional materials. A wide range of materials has been investigated for 3D printing applications, including polymer-metal composites [9] and polymer-ceramic composites [10], with the latter being particularly attractive due to their ease of processing, low cost, and versatility. However, commonly used polymers, such as petroleum-derived polypropylene (PP) and polystyrene (PS), are not only unsustainable in terms of their sources but also pose significant environmental concerns due to their non-biodegradable nature, leading to long-term environmental pollution and waste accumulation [11]. Therefore, biodegradable polymers derived from renewable resources are highly desirable as alternative materials for 3D printing applications [12].

PLA is a thermoplastic aliphatic polyester composed of repeating units derived from lactic acid. In general, PLA has gained attention due to its properties, such as non-toxicity, biocompatibility, and bio-derived origin, making it biodegradable. However, in terms of biodegradability, PLA is biodegradable through hydrolysis under composting conditions at 50–60 °C in high-humidity soil or marine environments [13]. Recently, PLA has been widely used as a 3D printing material over the past decade [14]. However, its widespread application remains limited by insufficient mechanical performance and poor thermal stability [15]. Especially in the field of environmental remediation, the limited functionality and inherently hydrophobic nature of PLA make it unsuitable for direct use as an adsorbent material. To overcome these limitations, various modification strategies have been explored, including surface functionalization, blending with hydrophilic polymers, and the incorporation of functional fillers [16]. Among these strategies, the incorporation of functional fillers is considered a particularly effective and practical approach, as it enables the simultaneous enhancement of mechanical and thermal properties while introducing active functional groups, all through relatively simple, cost-effective, and scalable processing methods [17].

Common reinforcing fillers for PLA include carbon fibers [18] and glass fibers [19]. These materials effectively enhance the mechanical strength, stiffness, and thermal stability of PLA, improving its durability during use [20]. However, their contribution to adsorption performance is limited due to the absence of active functional groups and their relatively inert surfaces. In addition, their non-biodegradable nature not only raises environmental concerns but also increases production costs [21]. To address these limitations, fillers with a bio-origin and, more importantly, a high density of functional groups are highly desirable. In our previous work, activated carbon (AC) derived from durian husk was successfully incorporated into 3D-printed PLA filaments, leading to improved mechanical properties and effective methylene blue (MB) adsorption [22], demonstrating the feasibility of agricultural waste as a functional filler. Recently, 3D-printed adsorbent materials have attracted considerable attention for environmental remediation applications owing to their ability to combine a customizable structure with functional adsorption performance. For instance, Lee et al. [23] developed 3D-printed ion-exchange materials with interconnected nanochannels, achieving significantly enhanced mass-transfer performance and rapid dye removal. In addition, Lu et al. [24] reported that adsorption performance is governed by the synergistic effects of ionic functionalities, pore structure, and mass-transfer properties, emphasizing the importance of both surface chemistry and structural design in adsorbent development. These studies highlight the growing research interest in functional adsorbent materials and emphasize the development of strategies to improve their performance across various applications, particularly in 3D-printing technologies. Motivated by this result, durian husk is further explored as a precursor for cellulose-based derivatives with abundant –COO– functional groups, such as carboxymethyl cellulose (CMC), and uses it as a filler for 3D-printed PLA filaments. Given its high cellulose content (ca. 60%) [25], it is a promising source for the production of carboxymethyl cellulose (CMC). Furthermore, CMC-based materials are known for their high hydrophilicity and strong adsorption capability, which are advantageous for environmental applications [26]. Therefore, incorporating CMC derived from durian husk into PLA filaments is a promising strategy for developing sustainable composites with enhanced adsorption performance.

In this work, cellulose was first extracted from durian husk and then used for the synthesis of CMC via etherification with MCA in the presence of varying amounts of NaOH. The as-synthesized CMC was subsequently used as a filler in 3D-printed filaments to produce PLA/CMC filaments via a single-screw extrusion technique. The effect of CMC content on the filaments was then characterized in terms of functional groups, surface morphology, thermal properties, swelling ratio, and mechanical properties. Finally, the filaments were applied as adsorbents for the removal of MB from water. The performance of filaments with different CMC contents in removing MB was evaluated, and the adsorption kinetics for each material were systematically investigated. Based on these results, the adsorption mechanism is also proposed.

## 2. Materials and Methods

### 2.1. Materials

Durian husk used in this study was obtained from a local durian farm in Chonburi province, Thailand. Sodium hydroxide (NaOH) was purchased from Carlo Erba Reagents S.r.l., Cornaredo, Italy, while monochloroacetic acid (MCA) was acquired from Loba Chemie Pvt. Ltd., Mumbai, India. Isopropanol was obtained from Qrec, Queenstown, New Zealand, and hydrochloric acid (HCl) was purchased from ANaPURE, Auckland, New Zealand. Commercial-grade poly(lactic acid) (PLA), an injection-molding grade under the trade name Ingeo 3100 HP (MW = 140,000 g mol^−1^), was supplied from NatureWorks LLC, Plymouth, MN, USA. All chemicals used in this study were of analytical grade and were used as received without further purification.

### 2.2. Extraction of Cellulose from Durian Husk

In a typical extraction, the durian husk waste was first cut into small pieces and washed with distilled water to remove surface contaminants, followed by drying in a hot-air oven at 120 °C for 24 h. The dried durian husk was then subjected to alkaline treatment using 10 wt.% NaOH at 120 °C for 3 h to remove hemicellulose and lignin components. After the alkaline treatment, the fibers were repeatedly rinsed with distilled water until a neutral pH was reached, followed by bleaching with 20 wt.% H_2_O_2_ at 80 °C for 24 h to eliminate lignin and other impurities. After the bleaching process, the fibers were thoroughly washed with distilled water to remove residual chemical reagents and then dried to obtain cellulose powder. The resulting powder was subsequently used for CMC synthesis, as described in the following section [27]. In this study, the cellulose extraction yield was calculated based on the dry weight of the obtained cellulose fibers (W_cellulose_) and the dry weight of the raw durian husk (W_dried durian_) according to Equation (1). Based on this calculation, the cellulose extraction yield obtained from durian husk was determined to be 21.01%.

(1) $$\%\;Yield\;\left(cellulose\right)=\frac{W_{cellulose}}{W_{dried\;durian}\;}\times 100$$

### 2.3. Synthesis of Carboxymethyl Cellulose (CMC)

In this work, CMC was prepared via an etherification reaction using the cellulose powder obtained from the experiment in the above section. Briefly, 5.0 g of cellulose powder was first dispersed in isopropanol, followed by the addition of NaOH at varying concentrations (20, 30, 40, 50, and 60 wt.%). The solution was stirred vigorously at room temperature for 30 min. Afterward, the mixture was heated to 55 °C, followed by the addition of 12.0 g of MCA (approximately 6.91 wt.%) with the molar ratio of cellulose: NaOH: MCA of 1:7.3:4.1. Then, the solution was continuously stirred for a further 1.5 h. The beaker was covered with aluminum foil and kept at 60 °C for 3 h. After the reaction was completed, the mixture was separated into solid and liquid phases. The solid phase was neutralized using acetic acid to remove residual alkali, then filtered and washed with ethanol three times to eliminate unreacted chemicals and by-products. Finally, it was dried at 60 °C overnight to obtain CMC powder [28]. The materials prepared using 20, 30, 40, 50, and 60 wt.% NaOH were denoted as CMC-20, CMC-30, CMC-40, CMC-50, and CMC-60, respectively. The percent yield of CMC was determined to be 84.67%, which was calculated based on the weight of the dried CMC powder (W_cmc_) and the weight of extracted cellulose fiber (W_cellulose_) according to Equation (2) [28].

(2) $$\%\;Yield\;\left(CMC\right)=\frac{W_{cmc}}{W_{cellulose}\;}\times 100$$

### 2.4. Estimation of the Degree of Substitution of CMC

The degree of substitution (DS) of CMC was evaluated using an acid-base titration method (ASTM D1439-Standard Test Methods for Sodium Carboxymethylcellulose), as described by Aggeryd and Olin [29], which is commonly used for DS analysis of CMC. Briefly, 0.8 g of CMC powder was suspended in 15 mL of ethanol and continuously stirred at ambient temperature for 25 min. Subsequently, 1 mL of 2.0 M nitric acid (HNO_3_) was added to the mixture and heated with stirring for 15 min to convert the sodium carboxylate groups (–COONa) into the acid form (–COOH). The resulting solid was then collected by filtration and rinsed with ethanol to remove excess acid, followed by drying at 60 °C for 1 h.

For the titration step, 0.2 g of the CMC was dissolved in 50 mL of distilled water. Then, 5 mL of 0.3 M NaOH was added, and the solution was heated to boiling for 15 min to ensure complete reaction. After cooling to room temperature, 2–3 drops of phenolphthalein indicator were added, and the solution was titrated with 0.3 M HCl until the colorless endpoint was reached. The volume of HCl consumed during titration was recorded and used to calculate the DS of CMC. The calculations were performed using Equations (3) and (4):

(3) $$A=\;\frac{(V_{NaOH}C_{NaOH}-V_{HCl}C_{HCl})\;}{m}$$

(4) $$\mathrm{DS}=\frac{0.162A}{1-0.058A}$$

where *A* represents the milliequivalents of acid consumed per gram of sample. *V*_NaOH_ and *C*_NaOH_ correspond to the volume (mL) and concentration (mol L^−1^) of NaOH solution added, respectively. Meanwhile, *V*_HCl_ and *C*_HCl_ refer to the volume (mL) and concentration (mol L^−1^) of HCl used during titration, while m represents the weight of the CMC (g). The constant 0.162 corresponds to the molecular weight of the anhydrous glucose unit (AGU) of cellulose (162 g mol^−1^), while 0.058 represents the net increase from the substitution of one hydroxyl group with a carboxymethyl group (58 g mol^−1^).

### 2.5. Fabrication by Single Screw Extruder

The PLA/CMC composite filaments were prepared via an extrusion process using a single-screw extruder. Firstly, moisture removal was carried out by drying PLA pellets and CMC powder in an oven at 60 °C for 12 h prior to processing and physically mixed with the different concentration of CMC-30, e.g., 0, 0.1, 0.5, and 1 wt.%, which then denoted as PLA, PLA/CMC-30 (0.1), PLA/CMC-30 (0.5), and PLA/CMC-30 (1.0). Then the PLA/CMC blends were fed into the feeder. Melt compounding and filament extrusion were performed using a single-screw extruder (Model SE-D50L20, CT Thailand, Samut Sakhon, Thailand) with a heated barrel and screw. The processing was controlled with barrel temperatures ranging from 160 to 220 °C, while the screw rotation speed was maintained at 25 rpm. The extruded filament was continuously cooled using an air-cooling system. The filament diameter was monitored and controlled to obtain a uniform diameter of 1.75 ± 0.05 mm. After cooling, the filament was collected and rolled onto the collector for use in the 3D printing.

### 2.6. Scanning Electron Microscope (SEM)

Scanning electron microscopy (SEM) was used to record the surface morphology of the as-prepared composite filaments. Prior to SEM analysis, the samples were cut into small pieces with dimensions of approximately 1 cm × 1 cm. To improve surface electrical conductivity, the samples were sputter-coated with a thin layer of gold on aluminum stubs. The surface morphology was observed using a JSM-IT300LV scanning electron microscope (JEOL, Tokyo, Japan) operated at an accelerating voltage of 15 kV.

### 2.7. Fourier Transform Infrared Spectroscopy (FTIR)

FTIR spectroscopy was performed to gain insight into the functional groups of the composite filament and to determine the interactions between the PLA polymer chains and CMC. The samples were prepared by first cutting the as-prepared composite filaments into small pieces and oven-drying them to eliminate residual moisture. FTIR spectra were then recorded using an FTIR spectrometer (FTIR-4100, INVENIOR, Bruker, Billerica, MA, USA) operating in attenuated total reflectance (ATR) mode over a wavenumber range of 4000–400 cm^−1^ with a resolution of 4 cm^−1^. For each sample, 64 scans were recorded under ambient conditions.

### 2.8. Differential Scanning Calorimetry (DSC)

The thermal behavior and degree of crystallinity of the as-prepared composite filaments were investigated using differential scanning calorimetry (DSC) analysis according to ASTM D3418 (Standard Test Method for Transition Temperatures and Enthalpies of Fusion and Crystallization of Polymers by Differential Scanning Calorimetry) [30]. For the measurement, the composite filaments were cut into small pieces, and approximately 5.0 mg of each sample was sealed in an aluminum DSC pan. The thermal program involved an initial heating stage from −50 to 220 °C, followed by a cooling step from 220 to 10 °C. Subsequently, the samples were reheated to 200 °C at a heating rate of 10 °C min^−1^ under a nitrogen purge of 50 cm^3^ min^−1^ to prevent oxidative degradation. All thermal data were recorded and analyzed by using NETZSCH analysis software (version Proteus thermal analysis 9.4.4). The degree of crystallinity (χ_c_) was calculated using Equation (5):

(5) $$\chi_{c}=\;\frac{{∆H}_{c}}{{∆H}_{m}^{0}\;\times \;w}$$

where ${∆H}_{c}\;$corresponds to the melting enthalpy of the specimen (J g^−1^), whereas ${∆H}_{m}^{0}\;$refers to the theoretical enthalpy of fusion for 100% crystalline PLA (93 J g^−1^) [31], and *w* refers to the weight fraction of PLA in the composite filament.

### 2.9. Swelling Ratio

The swelling ratio test was conducted to evaluate the water absorption behavior of the as-prepared composite filaments [32]. Briefly, dried filament samples were weighed to obtain the initial mass of dry weight (*W_d_*) prior to immersion in distilled water. The immersion times were varied at different times (0, 1, 3, 5, 15, 30, 45, 60, 90 and 120 min). After each period, the samples were taken out, wiped to remove excess surface water, and weighed to determine the swollen mass (*W_s_*). The swelling ratio (*SR*) was obtained using Equation (6):

(6) $$SR\;\left(\%\right)=\;\frac{W_{s}-\;W_{d}}{W_{d}}\times 100$$

where $W_{d}\;$represents the initial dry mass of the specimen, whereas $W_{s}$ corresponds to the mass of the swollen specimen at interval times.

### 2.10. Tensile Testing

The tensile properties of the composite filaments were evaluated using specimens prepared in accordance with ASTM D638 (Standard Test Method for Tensile Properties) [33]. Type IV specimens with dimensions of 30 mm × 10 mm × 0.4 mm were printed using a Raise3D Pro 2 Plus 3D printer equipped with a 0.4 mm diameter nozzle. Printing was carried out at a nozzle temperature of 200 °C, with the bed temperature maintained at 60 °C. The printing speed was maintained at 50 mm s^−1^, and the infill density was set to 50%. All specimens were printed in a flat (horizontal) orientation at a crosshead speed of 5 mm min^−1^. A raster angle of ±45 °C was used for all specimens. Tensile measurements were performed using a universal testing machine fitted with a 25 kN load cell.

### 2.11. Adsorption Test

In this work, the adsorption capability of the as-prepared composite filaments toward organic compounds was evaluated using MB dye as a model adsorbate [34]. In a typical adsorption experiment, the as-prepared composite filaments, i.e., PLA, PLA/CMC-30 (0.1), PLA/CMC-30 (0.5), and PLA/CMC-30 (1.0), were prepared into small pieces (ca. 0.5 cm × 0.5 cm), were added to test tubes containing 1.0 g of 50.0 ppm MB solution, which was defined as the initial dye concentration (*C*_0_). The mixture was then continuously agitated, and at predetermined times, i.e., 0, 15, 30, 60, 120, 180, 240, 300, 360, 420 min, the solution was collected for analysis to obtain *Ct*. UV–Vis spectroscopy was used to quantify the concentration of MB based on its absorbance at 665 nm. The percentage of the adsorbed MB was then calculated based on the peak change in MB concentration before and after adsorption, using the following equation (Equation (7)):

(7) $$\%\;\mathrm{adsorption}=\frac{C_{0}-C_{t}}{C_{0}}\times 100$$

where *C*_0_ represents the initial concentration of the dye, while *C_t_* corresponds to the concentration of MB at various times.

## 3. Results and Discussion

### 3.1. Characterizations

#### 3.1.1. DS of the As-Extracted CMC

**Figure 1:**
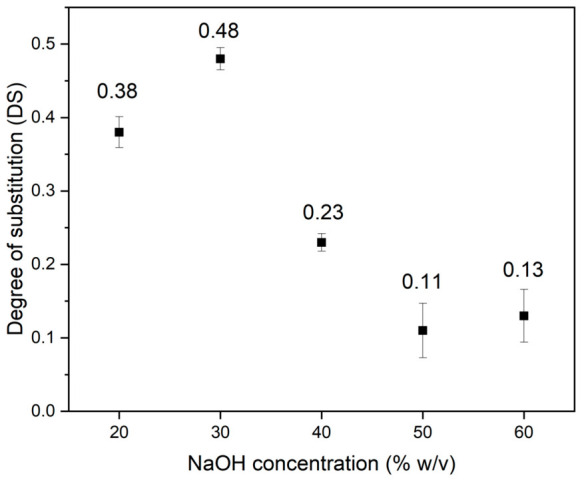
The DS of CMC prepared with different concentrations of NaOH.

As previously mentioned in Section 2, CMS was extracted from durian husk using different concentrations of NaOH, e.g., 20, 30, 40, 50, and 60 wt.%. To gain insight into the optimum NaOH concentration for the extraction process, the DS of each CMC sample was estimated. Using the same amount of MCA (12 g), as shown in Figure 1, the use of 20 wt.% NaOH (CMC-20) resulted in a DS of 0.38. This value increased to 0.48 when the NaOH concentration was raised to 30 wt.% (CMC-30). However, further increases in NaOH concentration to 40 (CMC-40), 50 (CMC-50), and 60 wt.% (CMC-60) led to a decrease in the DS value. This decline may be attributed to cellulose degradation under strongly alkaline conditions, which reduces both the molecular weight and crystallinity of the material. This alkaline degradation reduces the number of accessible and reactive hydroxyl (–OH) groups available for carboxymethyl substitution, leading to a lower DS value. Moreover, since NaOH can also react with MCA to form sodium glycolate, it is plausible that, at high NaOH concentrations, the reaction shifts toward the formation of sodium glycolate rather than CMC. This further decreases the extent of substitution along the cellulose chains [35]. This observation is consistent with previous studies on CMC derived from durian rind [36], mulberry paper waste [37], and sago waste [38]. In this work, CMC-30 was chosen for the preparation of PLA/CMC composite filament.

#### 3.1.2. Functional Groups

**Figure 2:**
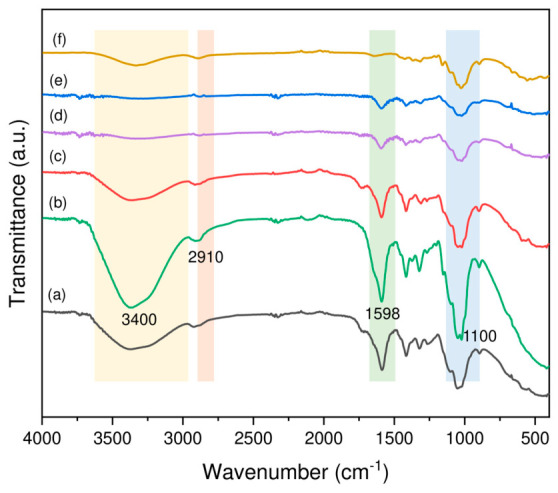
FTIR spectra of (**a**) CMC-20, (**b**) CMC-30, (**c**) CMC-40, (**d**) CMC-50, (**e**) CMC-60, and (**f**) cellulose.

Next, the functional groups of cellulose and CMC with different DS values were investigated using FTIR. As clearly depicted in Figure 2, cellulose exhibited typical absorption bands at 3200–3600, 2910, and 900–1100 cm^−1^, corresponding to O–H stretching, C–H stretching, and CH_2_ bending as well as C–O stretching, respectively [39]. After carboxymethylation, a new absorption peak appeared at around 1598 cm^−1^, which can be attributed to the carboxylate (–COO−) group. Another peak was also observed at around 1400–1480 cm^−1^, assignable to the carboxylate (–COO–Na^+^) groups [40]. Since these peaks were absent in the cellulose spectrum, this confirms the successful formation of carboxyl groups. Therefore, the absorption band observed at approximately 1750 cm^−1^ might be attributed to a partial amount of carboxylate groups (COOH) during the neutralization and purification steps. However, CMC samples prepared with higher NaOH concentrations showed a reduced intensity of the O–H stretching vibration. This reduction can be attributed to the disruption of hydrogen bonding caused by excessive alkalization. A similar decrease in O–H band intensity with increasing NaOH concentration has been reported for alkali-treated cellulose by Oh et al. [41].

**Figure 3:**
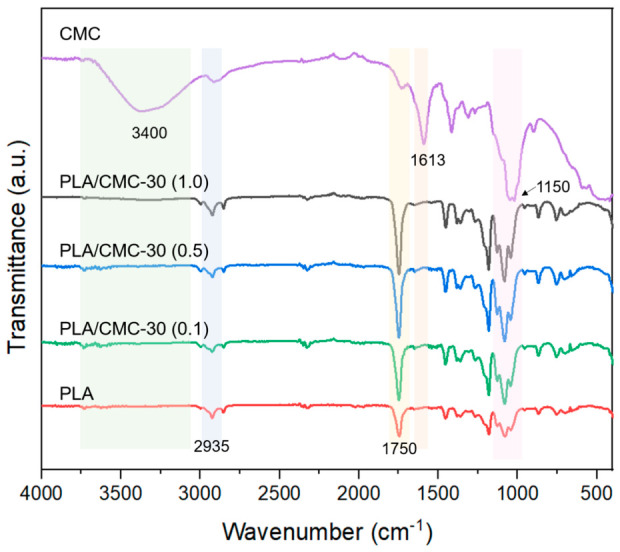
FTIR spectra of CMC-30, PLA filament, and PLA/CMC composite filaments prepared with different concentrations of CMC-30.

The FTIR spectra of CMC-20, PLA, and PLA/CMC composite filaments prepared with different CMC-20 concentrations (0, 0.1, 0.5, and 1 wt.%) are shown in Figure 3. As discussed above, CMC exhibited an absorption band at 3200–3600 cm^−1^, attributed to O–H stretching, and bands at 1750 and 1613 cm^−1^ corresponding to C=O and COO– stretching vibrations characteristic of the carboxymethyl cellulose structure, respectively. Meanwhile, the PLA filament showed typical absorption bands at 1750, 1100–1200, and 2990–2945 cm^−1^, attributable to the stretching vibrations of C=O, C–O–C, and C–H as well as CH_3_ groups, respectively [42]. After modification of PLA filaments with CMC, the characteristic absorption bands of PLA were largely preserved. However, the intensity of the O–H stretching band significantly decreased. This reduction is attributed to enhanced interfacial interactions between the CMC filler and the PLA matrix. A similar observation has been reported previously by Kamarudin et al. [43]. Overall, these results indicate that no new functional groups were formed between PLA and CMC, as no additional characteristic peaks were detected in the FT-IR spectra of the composite filaments compared with PLA.

#### 3.1.3. Surface Morphology

**Figure 4:**
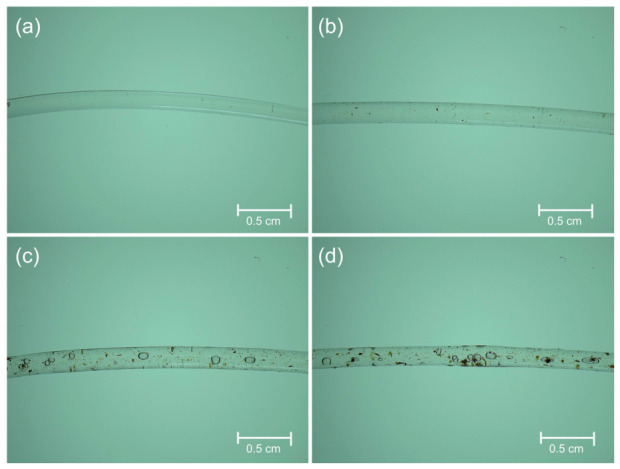
Optical microscopy images of (**a**) PLA, (**b**) PLA/CMC-30 (0.1), (**c**) PLA/CMC-30 (0.5), and (**d**) PLA/CMC-30 (1.0).

**Figure 5:**
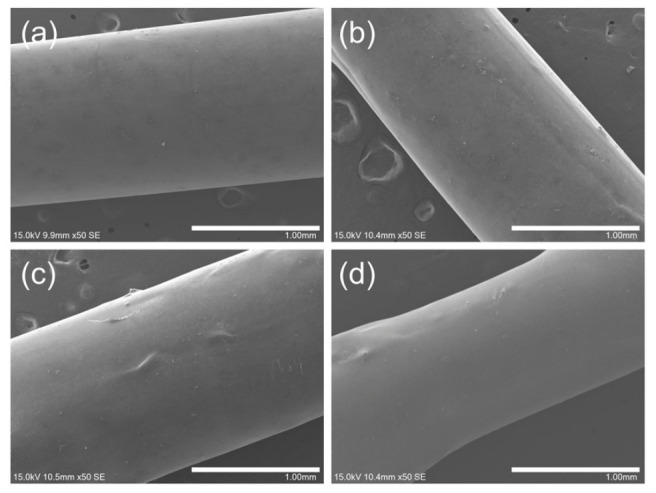
SEM images of (**a**) PLA, (**b**) PLA/CMC-30 (0.1), (**c**) PLA/CMC-30 (0.5), and (**d**) PLA/CMC-30 (1.0).

Next, the surface morphology of the as-prepared PLA/CMC composite filaments was examined using optical microscopy (Figure 4) and SEM (Figure 5) to evaluate both physical appearance and structural uniformity. Optical microscopy images showed that all filaments, including PLA/CMC-30 (0.1), PLA/CMC-30 (0.5), and PLA/CMC-30 (1.0), exhibited a clear and uniform internal structure with no observable surface defects. The only difference was that CMC-30 particles (appearing as dark regions in the filaments) became more pronounced with increasing CMC-30 content (Figure 4). These defects might be attributed to partial degradation of CMC during the extrusion process, leading to the formation of dark spots within the filament.

Meanwhile, SEM images showed that PLA/CMC-30 (0.1), PLA/CMC-30 (0.5), and PLA/CMC-30 (1.0) filaments exhibited an average diameter of approximately 0.75 mm and possessed relatively smooth and continuous surfaces without obvious cracks or defects (Figure 5). It is also evident that, although surface irregularities were occasionally detected in the PLA/CMC filaments, no significant particle agglomeration was observed on the surface. Therefore, it can be concluded that the incorporation of CMC-30 up to 1.0 wt.% did not lead to significant changes in the surface morphology of the filaments, indicating relatively good dispersion of CMC within the PLA matrix. This is consistent with previous reports by Liu et al. on CMC hydrogel fibers [44].

#### 3.1.4. Thermal Properties

**Figure 6:**
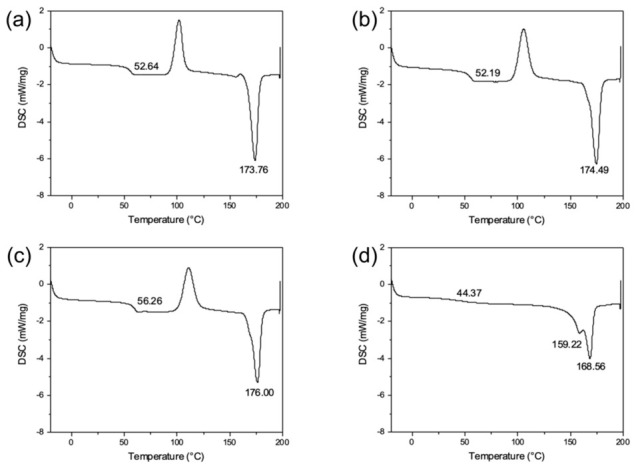
DSC thermograms of (**a**) PLA, (**b**) PLA/CMC-30 (0.1), (**c**) PLA/CMC-30 (0.5), and (**d**) PLA/CMC-30 (1.0) filaments.

Figure 6 shows the DSC thermograms of PLA, PLA/CMC-30 (0.1), PLA/CMC-30 (0.5), and PLA/CMC-30 (1.0) composite filaments, with detailed data presented in Table 1. The PLA filament exhibited a glass transition temperature (*T_g_*) of 52.64 °C and a melting temperature (*T_m_*) of 173.76 °C (Figure 6a, Table 1, Entry 1). The *T_g_* and *T_m_* values remained nearly unchanged after incorporation of 0.1 and 0.5 wt.% of CMC-30 (Figure 6b,c, Table 1, Entries 2 and 3). Notably, the *T_g_* values of PLA/CMC-30 (0.1) and PLA/CMC-30 (0.5) were 52.19 and 56.26 °C, respectively, while the corresponding *T_m_* values were slightly increased to 174.49 and 176.00 °C. These results clearly suggest that, at low filler loadings, CMC-30 has a minimal effect on the segmental mobility and thermal transitions of the PLA matrix. The slight increase in *T_m_*, on the other hand, may indicate a marginal enhancement in stability, possibly due to a weak nucleating effect of the dispersed CMC-30 particles. In contrast, a relatively significant reduction in *T_g_* was observed when 1.0 wt.% CMC-30 was incorporated, decreasing from 52.64 to 44.37 °C. This significant decrease suggests increased chain mobility, likely due to the disruption of intermolecular interactions among PLA chains at higher filler loadings. At this concentration, CMC-30 may act as a physical barrier that interferes with chain packing and reduces the effectiveness of intermolecular forces, thus facilitating segmental motion. In addition, two *T_m_* values were observed for PLA/CMC-30 (1.0), at 159.22 and 168.56 °C. The appearance of multiple melting peaks indicates the presence of heterogeneous crystalline structures, which may arise from uneven dispersion of CMC-30 or phase separation within the matrix. This behavior is commonly associated with limited compatibility or immiscibility between PLA and CMC [45], leading to the formation of regions with different crystal perfection or lamellar thickness.

However, the degree of crystallinity (*X_c_*) of PLA was 38.06%, whereas PLA/CMC-30 (0.1) and PLA/CMC-30 (0.5) showed decreases to 33.56 and 31.42% (Table 1, Entries 2 and 3), respectively, indicating that the addition of CMC interferes with the arrangement of PLA chains. This reduction can be attributed to limited compatibility between PLA chains and CMC-30 particles, which disrupts the regular packing of PLA molecular chains and consequently decreases crystallinity [46]. In contrast, PLA/CMC-30 (1.0) showed a slight increase in crystallinity (39.17%) compared to PLA (Table 1, Entry 4), which may be attributed to the higher CMC content providing additional heterogeneous nucleation sites that promote crystal formation [47]. Overall, these results suggest that the interfacial interactions between PLA and CMC significantly influence the crystallinity and thermal properties of the composite filaments.

#### 3.1.5. Swelling Ratio

**Figure 7:**
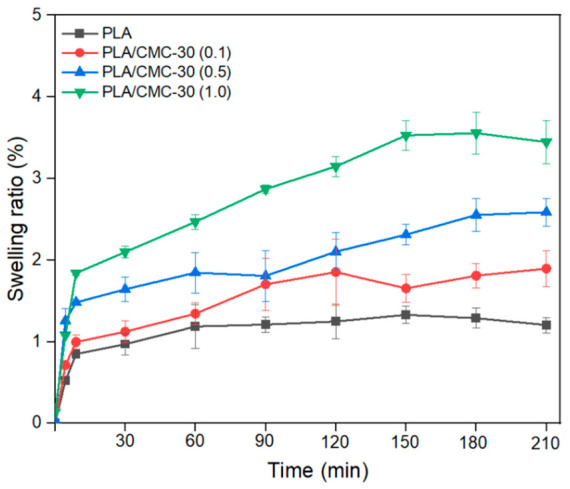
Water absorbency of PLA, PLA/CMC-30 (0.1), PLA/CMC-30 (0.5), and PLA/CMC-30 (1.0).

The water absorbency of PLA, PLA/CMC-30 (0.1), PLA/CMC-30 (0.5), and PLA/CMC-30 (1.0) was examined by swelling tests, and the results are shown in Figure 7. As clearly depicted, unmodified PLA exhibited the lowest swelling ratio, reaching approximately 1.2% after 210 min. This limited water absorption can be attributed to the hydrophobic nature of PLA, which restricts interactions between the polymer chains and water molecules. In contrast, the incorporation of CMC significantly enhanced the swelling behavior of the composite filaments, reaching approximately 1.9, 2.6, and 3.4% after 120 min for PLA/CMC-30 (0.1), PLA/CMC-30 (0.5), and PLA/CMC-30 (1.0), respectively. The results indicate that the swelling ratio increased upon the addition of CMC particles, as CMC is more hydrophilic than PLA, and increasing the CMC content enhances the interaction with water molecules [48]. As a result, PLA/CMC-30 (1.0) showed the highest swelling ratio, followed by PLA/CMC-30 (0.5) and PLA/CMC-30 (0.1), respectively, while unmodified PLA exhibited the lowest swelling ratio. These results confirm that higher CMC content leads to increased water absorbency and swelling capacity of the composite filaments. These results are in agreement with the DSC analysis, which exhibited a decrease in crystallinity, thereby allowing water to penetrate the composite structure and resulting in a higher swelling ratio.

#### 3.1.6. Mechanical Properties

**Figure 8:**
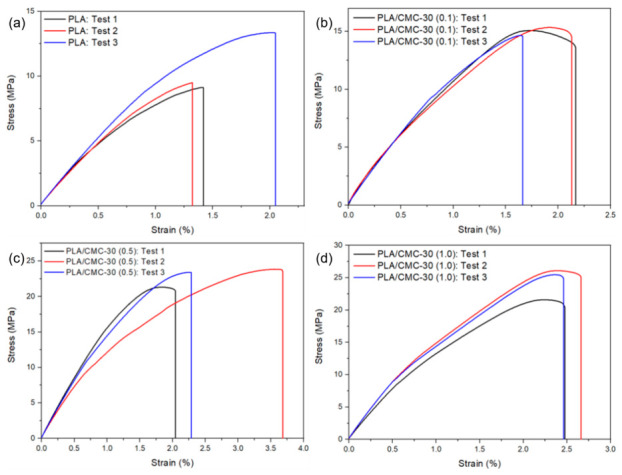
Stress–strain curve of (**a**) PLA, (**b**) PLA/CMC-30 (0.1), (**c**) PLA/CMC-30 (0.5), and (**d**) PLA/CMC-30 (1.0).

The mechanical properties of the composite filaments were evaluated using tensile testing following the ASTM D638 Type IV standard. Figure 8 and Table 2 summarize the tensile strength and elongation at break of the PLA, PLA/CMC-30 (0.1), PLA/CMC-30 (0.5), and PLA/CMC-30 (1.0). Prior to modification with CMC-30, PLA exhibited a tensile strength of 10.66 ± 2.34 MPa and an elongation at break of 1.58%, indicating the brittle nature of PLA. The incorporation of CMC-30 was observed to improve the mechanical performance, i.e., tensile strength increased with increasing CMC-30 content. In this case, the tensile strengths of PLA/CMC-30 (0.1), PLA/CMC-30 (0.5), and PLA/CMC-30 (1.0) were 15.03 ± 0.36, 22.86 ± 1.35, and 24.39 ± 2.43 MPa, respectively. A similar trend was observed in the elongation at break, indicating enhanced ductility of the composites compared to neat PLA. This improvement can be attributed to the CMC particles acting as a reinforcing phase within the PLA matrix, contributing to improved mechanical stability of the composite. c Overall, the addition of CMC effectively enhances both the strength and flexibility of PLA filaments, demonstrating its potential as an additive for improving composite filaments. A similar behavior in the mechanical properties of PLA-based composites has been reported by Choi et al. [49], where the addition of carboxymethyl cellulose affected the tensile properties of PLA composites due to the good distribution of CMC within the PLA matrix, and CMC enhances interfacial interactions between the components.

### 3.2. Adsorption Test

#### 3.2.1. Effect of Adsorption Time

**Figure 9:**
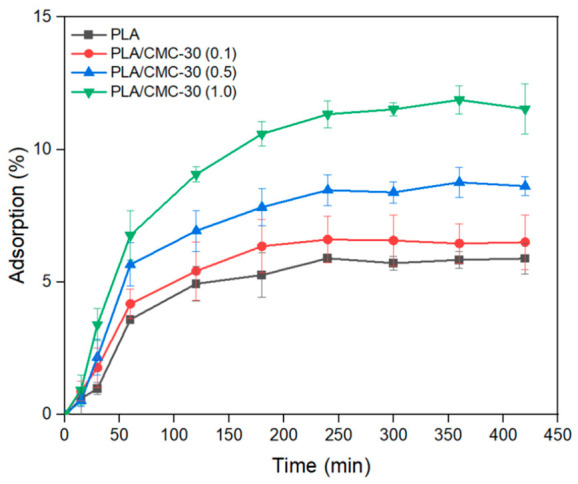
MB adsorption over PLA, PLA/CMC-30 (0.1), PLA/CMC-30 (0.5), and PLA/CMC-30 (1.0). Adsorption conditions: composite filaments (ca. 0.5 cm × 0.5 cm), 1 g; MB, 1.0 g (50.0 ppm); agitation time, 30–420 min; at room temperature.

In this work, the as-prepared composites were evaluated for their potential performance as adsorbents. MB dye was selected as a model pollutant, as it is commonly found in industrial wastewater, particularly in effluents from the textile, printing, leather, and paper industries [50]. As shown in Figure 9, unmodified PLA exhibited the lowest adsorption performance, removing only 6% of MB after 420 min. The incorporation of CMC significantly enhanced the adsorption capacity of the composite filaments. In this case, PLA/CMC-30 (0.1), PLA/CMC-30 (0.5), and PLA/CMC-30 (1.0) showed improved adsorption efficiencies, reaching approximately 6.6, 8.6, and 11.5% after 420 min, respectively. The low performance of unmodified PLA is owing to its hydrophobic nature and its smooth, relatively chemically inert surface. Although PLA contains ester groups (–COO–) that can participate in weak interactions such as hydrogen bonding, it cannot strongly bind cationic dyes such as MB (MB). Moreover, the presence of –CH_3_ groups renders the surface more hydrophobic, further reducing interactions with water and polar molecules. As a result, the interaction between PLA and MB molecules is weak, leading to limited adsorption capacity. The incorporation of CMC significantly enhanced the adsorption performance. In this case, the higher the CMC loading, the greater the percentage of MB adsorbed. This improvement is owing to the hydrophilic nature of CMC and the presence of abundant oxygen-containing functional groups (e.g., –OH and –COO–) [51], which can promote stronger interactions with MB through hydrogen bonding and electrostatic attraction, as well as increase water uptake and dye diffusion within the composite structure. This result clearly indicates that the as-prepared composites are promising candidates for use as adsorbents for the removal of organic compounds from water. In addition, the overall adsorption efficiency of the PLA/CMC composites was lower than in our previous studies for PLA/activated carbon composites [22], which had a maximum methylene blue adsorption efficiency of approximately of 51%. This difference may be attributed to the highly porous structure and large surface area of activated carbon, which provide an accessible adsorption site. In contrast, the PLA/CMC composites exhibited fewer available adsorption sites, resulting in lower adsorption efficiency compared to the PLA/activated carbon composite.

#### 3.2.2. Kinetic Analysis

**Figure 10:**
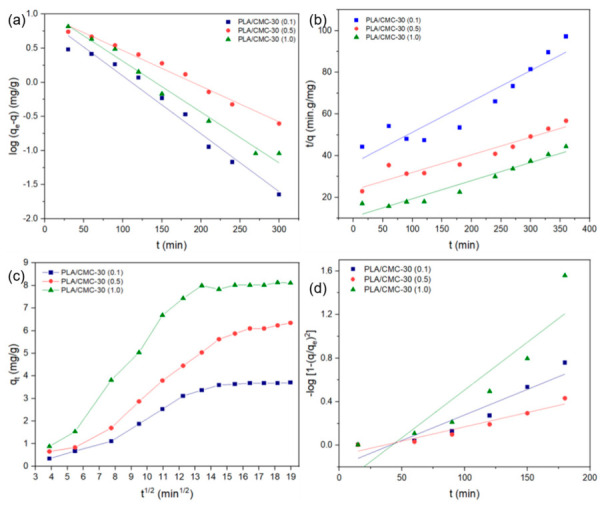
(**a**) Pseudo-first order, (**b**) Pseudo-second order, (**c**) intraparticle diffusion, and (**d**) Urano and Tachikawa kinetic plots for the adsorption of MB over PLA/CMC-30 (0.1), PLA/CMC-30 (0.5), and PLA/CMC-30 (1.0).

In this work, to gain insights into the adsorption behavior and rate-controlling mechanisms of MB onto PLA/CMC-30 (0.1), PLA/CMC-30 (0.5), and PLA/CMC-30 (1.0), the experimental data are shown in Figure 10. were first fitted to pseudo-first-order and pseudo-second-order models (Figure 10a,b and Table 3). The pseudo-first-order model, originally proposed by Lagergren S [52], has a linear form that can be expressed as follows:

(8) $$\log\left(q_{e}-q_{t}\right)=\log q_{e}-\;\frac{k_{1}}{2.303}\;t$$

where *q_t_* (mg g^−1^) and *q_e_* (mg g^−1^) represent the adsorption capacity at time (*t*) and at equilibrium, respectively, and *k*_1_ (min^−1^) is the rate constant of the pseudo-first-order model that can be determined from the slope of a linear plot of log(*q_e__−_ q_t_*) and *t*. Meanwhile, the pseudo-second-order model, commonly associated with chemisorption mechanisms [53], has a linear form that can be expressed as (Equation (9)):

(9) $$\frac{t}{q}=\left(\frac{1}{k_{2}q_{e}^{2}}\right)+\left(\frac{1}{q_{e}}\right)$$

where *q_t_* (mg g^−1^) and *q_e_* (mg g^−1^) represent the adsorption capacity at time (*t*) and at equilibrium, respectively. While *k*_2_ and *h* represent the pseudo-second-order rate constant (g mg^−1^ min^−1^) and initial adsorption rate (mg g^−1^ min^−1^), respectively, the values of *k*_2_ and *h* can be determined from the slope and intercept obtained by plotting between *t/q* and *t* (Equation (10)).

(10) $$h=k_{2}q_{e}^{2}$$

As shown in Figure 10a,b. and Table 3, the adsorption of MB followed the pseudo-first-order kinetic model for all materials, i.e., PLA/CMC-30 (0.1) (*k*_1_ = 0.020 min^−1^, R^2^ = 0.975), PLA/CMC-30 (0.5) (*k*_1_ = 0.012 min^−1^, R^2^ = 0.985), and PLA/CMC-30 (1.0) (*k*_1_ = 0.017 min^−1^, R^2^ = 0.986). The good agreement with the pseudo-first-order model suggests that the adsorption rate is primarily governed by the number of available adsorption sites and is dominated by physisorption processes, such as van der Waals interactions and diffusion-controlled mass transfer, rather than strong chemical bonding. This indicates that the adsorption of MB onto PLA/CMC composites is primarily governed by physical adsorption and diffusion mechanisms [54]. This behavior can be attributed to the hydrophobic nature of PLA, which limits its interaction with hydrophilic and cationic MB molecules [55]. The adsorption over PLA/CMC-30 (0.1) showed the highest *k*_1_ value (0.020 min^−1^), which might be due to the material possessing the highest dispersion of CMC compared to the other two, resulting in greater accessibility of functional groups. However, increasing the CMC content to 0.5 wt.% decreased the *k*_1_ value to 0.012 min^−1^, likely due to aggregation of the loaded CMC, which reduced accessibility to the –COO– groups. Surprisingly, further increasing the CMC content to 1 wt.% increased the *k*_1_ value to 0.017 min^−1^. This may suggest that although CMC particles are aggregated, the higher loading of CMC results in a greater number of exposed –COO– groups available for the adsorption of MB [56].

Next, the diffusion mechanism involved in the adsorption process was examined using the intraparticle diffusion model proposed by Weber and Morris [57]. This model can be expressed as follows:

(11) $$q_{t}=\;k_{int\;}t^{1/2}+C$$

where *q_t_* (mg g^−1^) is the amount of adsorbate adsorbed at time, *k_int_* (mg L^−1^ min^–1/2^) is the intraparticle diffusion rate constant, and C (mg g^−1^) is the intercept related to the thickness of the boundary layer. If the plot between *q_t_* and *t*^1*/*2^ is linear and passes through the origin, intraparticle diffusion is considered the sole rate-limiting step. As shown in Figure 10c, the plots between *q_t_* and *t*^1*/*2^ for all PLA/CMC composites exhibited multi-linear behavior, and none of the lines pass through the origin, indicating that intraparticle diffusion is not the rate-limiting step. The adsorption process can be described by two stages, particularly an initial rapid phase attributed to external mass transfer [58], followed by a slower stage corresponding to diffusion within the internal structure of the adsorbent. At longer contact times, the curves approach equilibrium due to the saturation of available sites [59]. These results suggest that the overall adsorption is governed by a combination of external diffusion and intraparticle transport mechanisms rather than a sole rate-limiting step [60].

To further evaluate the multistep nature of the adsorption process, the kinetic data were analyzed using the Urano and Tachikawa model [61]. In this approach, the kinetic data were linearized by plotting −log [1 − (*q/q_e_*)^2^] versus *t*, as shown in Figure 10c. A linear relationship passing through the origin generally indicates that intraparticle diffusion is the sole rate-limiting step. However, all PLA/CMC filaments exhibit relatively linear trends, and none of the plots intersect the origin. This behavior indicates that, although intraparticle diffusion contributes to the adsorption process, it is not the only controlling mechanism. Instead, the overall process is influenced by multiple concurrent mechanisms, including external mass transfer and interactions between MB molecules and the functional groups present on the adsorbent surface [62]. Consequently, the adsorption behavior is better described as a combined diffusion-surface-controlled process rather than a single rate-limiting step [63].

## 4. Conclusions

In summary, we have successfully synthesized CMC with a DS of 0.48 from durian husk-derived cellulose via etherification with MCA in the presence of 30 wt.% NaOH. The extracted CMC was also successfully incorporated into 3D-printed PLA filaments using a single-screw extrusion technique to produce PLA/CMC filaments. Characterization results showed that the as-prepared filaments not only exhibited enhanced thermal and mechanical properties but also possessed carboxylate (–COO–) groups from CMC, making them suitable as an adsorbent for the removal of MB in water. Over PLA/CMC-30 (0.1), MB removal followed a pseudo-first-order kinetic model, with a removal rate constant (*k*_1_) of 0.020 min^−1^. Overall, this work demonstrates that PLA/CMC filaments derived from agricultural waste offer a sustainable and multifunctional material with enhanced performance for environmental remediation applications.

## Figures and Tables

**Table 1:** Thermal properties of PLA, PLA/CMC-30 (0.1), PLA/CMC-30 (0.5), and PLA/CMC-30 (1.0).

Entry	Sample	*T_g_* (°C)	*T_m_* (°C)	△*H_m_*	*X_c_* (%)
1	PLA	52.64	173.76	35.40	38.06
2	PLA/CMC-30 (0.1)	52.19	174.49	31.21	33.56
3	PLA/CMC-30 (0.5)	56.26	176.00	29.22	31.42
4	PLA/CMC-30 (1.0)	44.37	159.22, 168.56	36.43	39.17

**Table 2:** Detailed data of the stress–strain curves of PLA, PLA/CMC-30 (0.1), PLA/CMC-30 (0.5), and PLA/CMC-30 (1.0).

Sample	Tensile Strength (MPa)	Elongation at Breaks (%)
PLA	10.7 ± 2.3	2 ± 0.38
PLA/CMC-30 (0.1)	15.0 ± 0.4	2 ± 0.15
PLA/CMC-30 (0.5)	22.9 ± 1.4	3 ± 0.91
PLA/CMC-30 (1.0)	24.4 ± 2.4	2 ± 0.09

**Table 3:** Kinetic data for the adsorption of MB over PLA/CMC-30 (0.1), PLA/CMC-30 (0.5), and PLA/CMC-30 (1.0).

Kinetic Model	Parameter	PLA/CMC-30 (0.1)	PLA/CMC-30 (0.5)	PLA/CMC-30 (1.0)
Pseudo-first order	*k*_1_ (min^−1^)	0.020	0.012	0.017
	*R* ^2^	0.975	0.985	0.986
Pseudo-second order	*k*_2_ × 10^−4^ (g mg^−1^ min^−1^)	6.024	3.038	7.124
	*h* (mg g^−1^ min^−1^)	0.027	0.043	0.094
	*R* ^2^	0.886	0.916	0.942

## Data Availability

The data that support the findings of this study are available from the corresponding author upon reasonable request.
